# Enhanced identification of Group B streptococcus in infants with suspected meningitis in Ethiopia

**DOI:** 10.1371/journal.pone.0242628

**Published:** 2020-11-19

**Authors:** Alene Geteneh, Tesfaye Kassa, Yared Alemu, Derbie Alemu, Mulugeta Kiros, Henok Andualem, Workeabeba Abebe, Tinsae Alemayehu, Dawit Hailu Alemayehu, Adane Mihret, Andargachew Mulu, Wude Mihret

**Affiliations:** 1 Department of Medical Laboratory Science, College of Health Science, Woldia University, Woldia, Ethiopia; 2 School of Medical Laboratory Science, Jimma University, Jimma, Ethiopia; 3 Arba Minch College of Health Science, Arba Minch, Ethiopia; 4 Department of Medical Laboratory Science, College of Health Science, Debre Tabor University, Debre Tabor, Ethiopia; 5 School of Medicine, Addis Ababa University, Addis Ababa, Ethiopia; 6 Armauer Hanson Research Institute, Addis Ababa, Ethiopia; Public Health England, UNITED KINGDOM

## Abstract

Meningitis is one of the top ten causes of death among Ethiopian infants. Group B streptococcus (GBS) has emerged as a leading cause of meningitis in neonates and young infants, resulting in high mortality. Despite this, there is no report on GBS associated meningitis in Ethiopia where infant meningitis is common. Hence, the aim of this study was to determine the proportion of GBS associated meningitis among Ethiopian infants. PCR was prospectively used to detect GBS in culture-negative cerebrospinal fluid (CSF) samples, which were collected from infants suspected for meningitis, at Tikur Anbessa specialized hospital, Ethiopia, over a one-year period. GBS was detected by PCR in 63.9% of culture-negative CSF samples. Out of the 46 GBS positive infants, 10.9% (n = 5) of them died. The late onset of GBS (LOGBS) disease was noted to have a poor outcome with 3 LOGBS out of 5 GBS positive samples collected from patients with the final outcome of death. PCR was advantageous in the identification of GBS in culture-negative CSF samples. GBS was detected in 64% of the CSF samples from infants with meningitis compared with zero-detection rate by culture.

## Introduction

Bacterial meningitis (BM) is the leading cause of morbidity and mortality in infants [1]. There are many bacterial etiologic agents of BM in this age group [1, 2]. Among these, GBS (also known as S*treptococcus agalactiae*) and *Escherichia coli* are the predominant causes of illness. Although they are the major causes of death in infants, there is no any approved vaccine available for these pathogens yet [3]. About 20–30% of healthy women carry GBS on their vaginal or rectal mucosa [4]. This maternal colonization is the primary means for newborn GBS acquisition either via ascending infection or during birth through the infected birth canal as neonates aspirate contaminated amniotic or vaginal fluids [5]. The invasive GBS infections can be categorized into early-onset disease (EOD), late-onset disease (LOD), and rarely as ultra-late onset disease (ULOD). The EOD occurs within the first six days of life while the LOD and the ULOD occurs from 7 up to 89 days, and after 3 months of age respectively [6, 7]. Recently, GBS has emerged as a leading cause of meningitis in neonates and young infants, resulting in high mortality [8]. However, GBS is not still considered as the major bacterial etiological agent of infant sepsis and meningitis in most of the developing countries including Ethiopia [9]. Thus, this study was aimed to determine the proportion of GBS associated meningitis among Ethiopian infants using PCR. The data generated from this study will used by policymakers to better understand the epidemiology of the disease in the local setting and set disease prevention and control strategies.

### Study setup

An institutional based cross-sectional study was implemented from June to October 2018 at TASH, Addis Ababa, in collaboration with Armauer Hanson Research Institute (AHRI). The target population in this study were those infants under 1 year of age from TASH who were clinically suspected of meningitis (infants with sudden onset of fever, meningeal irritation, or altered consciousness). A total of 72 infants aged from one day to one year who had a CSF sample available (≥200ul) and were volunteer to participate (parents/guardian`s consented) were consecutively included. CSF white cells count, protein, glucose, and patient outcome were obtained from the patient’s record and from other secondary sources.

### Laboratory procedure

The CSF samples, collected under aseptic conditions were submitted to the microbiology laboratory of TASH and processed within 1 hour after collection [10]. The macroscopic appearances of the samples were noted as clear, turbid yellow, or bloody. CSF protein was measured using a Mindray machine (Mindray, Shenzhen-China), while CSF glucose was measured using Sysmex. After the above-mentioned parameter measurement, the CSF was centrifuged at 1000g for 10 to 15 minutes. Gram staining was then done on the CSF sediment. Subsequently, the CSF sediment was inoculated into brain heart infusion (BHI) broth, chocolate and blood agar plates (both incubated with 5% CO2), and MacConkey (in aerobic condition). All the broth and agar plates were then incubated at 35–37°C for up to 72hrs. The BHI broth was incubated under the shaker incubator at 37°C for up to 72hrs so as facilitate microbial growth in aerobic conditions [10]. The remaining CSF samples were used to extract the genomic DNA using Qiagen DNA mini kit (QIAamp DNA mini kit, Hilden, Germany) according to the manufacturer’s instruction.

Since the CAMP factor is a major virulence determinant region among most GBS serotypes, we used a primer that targets the gene encoding CAMP factor (*cfb*) (Sequence ID: MK134700.1). The reaction mixture (25μl) contained: 12.5 μl HotStarTaq mix, 1.5 μl (5uM) of each of SAG 59 (TTTCACCAGCTGTATTAGAAGTA) and SAG 190 (GTTCCCTGAACATTATCTTTGAT) primers, 4.5 μl molecular grade water, and 5 μl DNA template. The thermal cycling was 94°C for 12 minutes, followed by 40 cycles of amplification (94°C for 30 seconds, 55°C for 30 seconds and 72°C for 30 seconds). A final extension step was carried out at 72°C for 2 minutes. Gel electrophoresis was run on 2.0% agarose gel for visualizing the amplified PCR products/the target bands using the 100bp DNA ladder as the reference marker and DNA of GBS (ATCC 12386) as a positive control with 153bp amplicon size [11, 12].

### Quality control

In each laboratory work (culture and PCR run), both positive and negative controls were run in parallel to make sure the test was free from false positive and false-negative results. Before and after each master mix preparations, the biosafety cabinet was cleaned with 10% bleach and with 70% alcohol to prevent any contamination. To prevent any cross-contamination, each laboratory work (DNA extraction, master mix preparation, template addition, and sample storage) was performed in different rooms.

### Ethical consideration

Ethical clearance was obtained from an institutional review board of Jimma University with a reference number IHRPGD/143/2018. Written informed consent was also obtained from infants' parents or guardians for use of their infants leftover CSF to this study. Study participants' right to refuse and not give CSF samples without affecting their routine medical services were granted. Findings were immediately communicated with pediatricians at TASH to help infants if still admitted and to take into consideration the result on which segment of infants was burdened with GBS.

## Results

The majority of the study participants, 61.1% (n = 44), were neonates (aged ≤ 28 days) and the remaining 38.9% (n = 28) of them were infants who are aged >28 days. The mean age at the onset of meningitis was 49.8 ± 83.6 days (1–365 days). The male to female proportion was nearly 1.7:1 i.e. 62.5% being male infants. Fever was one of the early clinical events expected to occur in most cases of meningitis. In this study, 43% (n = 31) of the total infants were observed to have a fever. Overall, vomiting or reduced ability to suck breastfeeding (n = 45), fever (n = 31), and altered consciousness (n = 27) were the most frequent clinical presentations noted in this study. Of the clinical presentations examined in infants; 61.3% (n = 19) were with fever, 48.1% (n = 13) with impaired consciousness, and 55.6% (n = 25) who have had vomiting or reduced ability to suck for breastfeeding were positive for GBS (Table 1). Most of the infants; 54/72 (75%), had a history of antibiotics use before the spinal tap procedure, which could decrease GBS culture recoverability. According to the clinical disease onset by age, most of the infants were classified under the LOD as observed by the proportion of 76.4% (55/72). While the proportion of EOD and the ULOD was 12.5% (9/72) and 11.1% (8/72) respectively.

**Table 1:** Bivariate analysis of demographic and clinical features of infants suspected with meningitis at TASH from June 2018 to Oct. 2019, Addis Ababa, Ethiopia.

Characteristics		GBS		COR (95%CI)	p-value
		Positive (%)	Negative (%)		
Age	< 7 days (n = 9)	4 (44.4)	5 (59.6)	0.27 (0.03,2.12)	0.211
	7–89 days (n = 55)	36 (65.5)	19 (34.5)	0.63 (0.12,3.44)	0.595
	≥ 90 days (n = 8)	6 (75)	2(25)	R	
Sex	Male (n = 45)	30(66.7)	15(33.3)	1.375 (0.51, 3.69)	0.526
	Female(n = 27)	16(59.7)	11(40.3)	R	
Fever*	Yes (n = 31)	19(61.3)	12(38.7)	0.82(0.31,2.16)	0.690
	No (n = 41)	27(65.9)	14(34.1)	R	
Impaired consciousness	Yes (n = 27)	13(48.1)	14(51.9)	0.34 (0.12,0.92)	0.034
	No (n = 45)	12(26.7)	33(73.3)	R	
Vomiting/reduced breastfeeding	Yes (n = 45)	25(55.6)	20(44.4)	0.36(0.12,1.05)	0.062
	No(n = 27)	21(77.8)	6(22.2)	R	
Neck stiffness	Yes (n = 2)	1(50)	1(50)	0.56(0.03,9.27)	0.682
	No(n = 70)	45(64.3)	25(35.7)	R	
CSF WCC^	<20 cells/μl (n = 43)	29(67.4)	14(32.6)	R	
	≥ 20 cells/μl (n = 11)	8(72.7)	3(27.3)	1.29(0.29, 5.61)	0.737
CSF glucose^	≤ 40mg/dl (n = 8)	6(75)	2(25)	1.26(0.21,7.65)	0.799
	> 40mg/dl (n = 27)	19(70.4)	8(29.6)	R	
CSF protein ^	≤ 100mg/dl (n = 30)	20(66.7)	10(33.3)	R	
	> 100mg/dl (n = 16)	10(62.5)	6(37.5)	0.83(0.24, 2.96)	0.778
Outcome of infants^	Recovery (n = 16)	5(31.3)	11(68.7)	R	
	Death (n = 6)	5(83.3)	1(16.7)	11.0(1.005,120.43)	0.050

* Fever = temp. ≥ 38 ^o^ c, unknown/no result = data not available on the patient’s medical record.

* Normal values: WCC = 0-5/μL, CSF Protein = 40–100 mg/dl and CSF glucose = 40–75 mg/dl [13, 14].

^ done with available data since this used secondary data from patients medical record.

The CSF white cells count, protein, and glucose concentrations were obtained for more than half of the infants. Unlike most criteria’s used to exclude bacterial meningitis, GBS was detected in more than 67.4% (n = 29) of infants without pleocytosis (as defined by CSF cells count of ≥ 20 cells /μl) in our study. Of the infants with available glucose concentration data, 8 (out of 35) had below 40 mg/dl, while infants with protein concentration data 16 (out of 46) had above 100mg/dl, as expected in bacterial meningitis cases.

Similarly, the appearance of CSF was noted and nearly 78% of all the samples were found to be clear. Most of the confirmed GBS cases in our study have presented with normal CSF parameters in terms of cell count, protein, and glucose concentrations (see Table 1). This could question the reliability of CSF parameters in the prognosis of BM. The overall magnitude of GBS in infants with suspected meningitis was 63.9% (46/72). Proportionally higher prevalence of GBS was noted in young infants aged > 7 days (Table 1). Having impaired consciousness and Outcome of infants (death) was statistically associated with GBS positivity.

Death was observed in 6 of the 72 enrolled study infants. Death might be attributed by GBS in 10.9% (5out of 46 confirmed GBS cases) meningitis cases. Four of the dead infants were in the age group less than or equal to 28 days, while one was 44 days old and the other one was 4 months old. Considering the rate of GBS positivity (GBS vs. no GBS), the probable death was assumed (10.9% (5/46) vs. 3.8% (1/26)).

## Discussion

In the present study, GBS was found high in infants with suspected meningitis cases, 63.9% (46/72), which is quite comparable with the study done in Korea 69% [15] and lower the USA 86.1% [16]. The finding was higher relative to the study carried out in China (46.5%) [17], in USA (53.6%) [18], in England (52%) [19], in South Africa (44%) [20] and in Malawi (26.3%) [8]. The difference observed may be due to the detection method utilized, maternal use of intra partum antibiotic prophylaxis (IAP), study setting differences, or population variations. Possibly inclusion of infants’ age beyond 3 months and the use of a more sensitive detection tool in our study might have contributed to the increment of the proportion of GBS detection.

Detection through cfb gene-targeted PCR was found to be too enhanced from all culture-negative results of GBS to 63.9%. Similar studies have also demonstrated the positivity of culture-negative CSF samples when tested with more sensitive techniques like PCR. Culture finding could be negative, possibly due to previous antibiotic exposure before CSF collection, the low number of the bacterium, or lower inoculum size. A comparative study in Ireland has shown the reputation of CSF PCR over culture. In contrast to culture which is a considered as a gold standard tool, 77.3% more detection capacity was offered by PCR [21]. In agreement to our study, other studies in Brazil (3.8% vs. 29.2%) [22], Iran (27.7% vs. 43.8%) [23], China (0% vs. 50.7%) [24] and Jimma, Ethiopia (3% vs. 33%) were also indicated the sensitivity of PCR over culture [25].

Late-onset GBS disease (65.5%) was observed high relative to EOGBS (44.4%) (Table 1). This study finding was relatively lower compared to results in China (74%) [26] and in the US (86.5%) [16]. High community or nosocomial acquisition, exposures to colonized parents and siblings, breastfeeding during maternal mastitis, and or prematurity could be the possible reasons [27, 28]. Although our sample size was too small, infants presented with LOGBS were more likely to have death outcomes, i.e. 3 out of 5 GBS positive deaths were due to LOGBS. Similar studies have shown that the LOGBS causes higher morbidity and mortality than EOGBS and ULOGBS at the time of discharge [27, 28]. A finding from Australia has verified that infants with LOD cases were 3 times more likely to develop neurodevelopmental sequelae or die in the course of admittance than ULOD, this reflects the vulnerability of infants in the first 90 days of life [28].

Disease presentations like fever was anticipated to occur in most cases of meningitis [29]. The current study noted vomiting or reduced ability to suck breastfeeding (n = 45), followed by fever (n = 31) and altered consciousness (n = 27) as the most frequent clinical presentations. Cerebrospinal fluid parameters such as protein, glucose, and white cell count (WCC) were used to assess the likely etiological agents, provide a protein concentration <60 mg/dl and WCC <90 cells/ μl were found to be optimal cut-offs for excluding bacterial meningitis [30]. Opposing this conclusion, the present study found more than 67% of infants positive for GBS had WCC < 20 cells/ μl. A similar study in India has also described that 16% of infants with GBS had 0–20 cells/ul [31], and in the USA nearly 11% of cases lacked CSF pleocytosis [18].

The probable mortality of GBS meningitis in the current study was 10.9% (5/46), lower compared with the study carried out in South Africa (18%) [20]. This might be due to the inclusion of all the invasive GBS infections (including sepsis and meningitis) in South Africa. The use of limited sample size, unavailability of data of some infants who are transferred out, lost with their appointment, and withdraw against medical advice, may have underestimated the real burden of mortality in the current study.

This study tried to address the magnitude of GBS meningitis in infants and gave an insight into how more sensitive tools would implicate for culture-negative suspected meningitis cases. However, the main constraint to this study was the use of limited sample size and the inability of our study to address potential risk factors such as mode of delivery, maternal colonization, maternal mastitis, birth gestational age, and birth weight as this study used secondary data regarded factors were not available.

## Conclusions

In this study, a high proportion of GBS was detected among infants suspected of meningitis at TASH, Addis Ababa, Ethiopia. Accordingly, GBS was found out to be the major etiologic agent of BM among neonates and young infants. The current study also showed that the onset of disease matters to the outcome of patients, indicating three out of five died infants had a late-onset presentation. In addition, the role of GBS can be underestimated by using culture detection techniques in contrast to molecular assays like PCR. This was observed by a high positivity rate (63.9%) of GBS among participants who were culture negative and a history of antibiotic exposure before the spinal tap. We recommend further studies of this kind with a larger sample sizes to be conducted so as to devise the basic prevention and control strategies.
